# Bipolar radiofrequency ablation for re-entrant ventricular tachycardia of right bundle branch block and left bundle branch block morphologies with the common slow conduction zone at the left ventricular summit: a case report

**DOI:** 10.1093/ehjcr/ytae191

**Published:** 2024-04-16

**Authors:** Masahiro Toba, Toshihiro Nasu, Nobuyoshi Nekomiya, Takao Makino, Hisashi Yokoshiki

**Affiliations:** Department of Cardiovascular Medicine, Sapporo City General Hospital, Kita-11, Nishi-13, Chuo-ku, Sapporo 060-8604, Japan; Division of Medical Engineering Center, Sapporo City General Hospital, Sapporo, Japan; Division of Medical Engineering Center, Sapporo City General Hospital, Sapporo, Japan; Department of Cardiovascular Medicine, Sapporo City General Hospital, Kita-11, Nishi-13, Chuo-ku, Sapporo 060-8604, Japan; Department of Cardiovascular Medicine, Sapporo City General Hospital, Kita-11, Nishi-13, Chuo-ku, Sapporo 060-8604, Japan

**Keywords:** Left ventricular summit, Ventricular tachycardia, Re-entry, Radiofrequency ablation, Bipolar ablation, Intramural substrate, Case report

## Abstract

**Background:**

The left ventricular (LV) summit has anatomical limitations, so the detailed mapping is difficult. Therefore, the mechanism of ventricular tachycardia (VT) originating from the LV summit is not well understood.

**Case summary:**

A 70-year-old man had VTs with right bundle branch block (VT1 and VT3) and left bundle branch block (VT2) morphologies originating from the left ventricular summit (LV summit). During the VT2 and VT3, fragmented potentials, which occurred earlier than the QRS onset, were recorded from bipolar electrodes of a catheter at the anterior intraventricular vein (AIV). By pacing from right ventricular apex, constant and progressive fusion were observed. During the entrainment pacing, the fragmented potentials in the AIV catheter were activated orthodromically and those in the His bundle were activated antidromically. In addition, there were two components of the ventricular electrogram at the LV summit area with the interval of more than 100 ms during the VTs. We performed bipolar radiofrequency ablation between the LV endocardium and AIV, and the VTs became non-inducible.

**Discussion:**

Non-sustained VT/premature ventricular contraction originating from LV summit is generally considered to occur due to abnormal automaticity or triggered activity. In contrast, using entrainment technique, we demonstrated that the VTs with multiple morphologies were sustained with a re-entrant mechanism. Fragmentated potentials recorded in the AIV catheter were activated orthodromically with the entrainment pacing, indicating the slowly conducting isthmus. The intramural VT substrate was also suggested with a prolonged conduction time between the two ventricular components during the VTs.

**Learning points**

- Most cases of ventricular arrhythmias originating from the left ventricular (LV) summit are considered to occur with a non-re-entrant mechanism, and the re-entrant mechanism has not been proved.
- Entrainment pacing helped to demonstrate a re-entry of the LV summit ventricular tachycardias (VTs) where the mapping was difficult with anatomical limitations.
- A prolong conduction time between two ventricular components during the VTs implied intramural substrate in the LV summit, so bipolar ablation was needed.

## Introduction

The left ventricular (LV) summit, the highest point of the LV epicardium, is a triangular region near the bifurcation of the left anterior descending and the left circumflex coronary arteries.^1^ Because of these anatomical limitations, the detailed mapping is difficult. Therefore, the mechanism of ventricular tachycardia (VT) originating from LV summit is not well understood. In this case, by entrainment technique, we revealed the re-entrant mechanism of the VTs with the slow conducting zone at the LV summit area.

## Summary figure

**Table:** 

Time line	
December 2013	He underwent percutaneous coronary intervention of left anterior descending artery due to effort angina pectoris.
June 2014	He was transferred for out-of-hospital cardiopulmonary arrest due to ventricular fibrillation. There was no significant stenosis of the coronary arteries.
July 2014	Implantable cardioverter-defibrillator was implanted.
April 2022	He was referred for palpitation with a ventricular tachycardia (VT) with left bundle branch block (LBBB) configuration and inferior axis deviation.
	We performed 1st radiofrequency catheter ablation (RFCA). Two types of VTs with LBBB and RBBB morphology originating from the LV summit were recognized.
	RF energy was delivered at the left coronary cusp, left ventricular outflow tract (LVOT), and right ventricular outflow tract.
November 2022	He was hospitalized for congestive heart failure.
	We performed coronary angiography, and there was no stenosis of the coronary arteries.
	The echocardiography showed no regional wall motion abnormality with a left ventricular ejection fraction of 56%.
December 2022	The VT with RBBB configuration recurred, and we performed 2nd RFCA.
	Bipolar radiofrequency ablation between anterior intraventricular vein and left ventricular endocardium was performed.

## Case presentation

**Figure 1:**
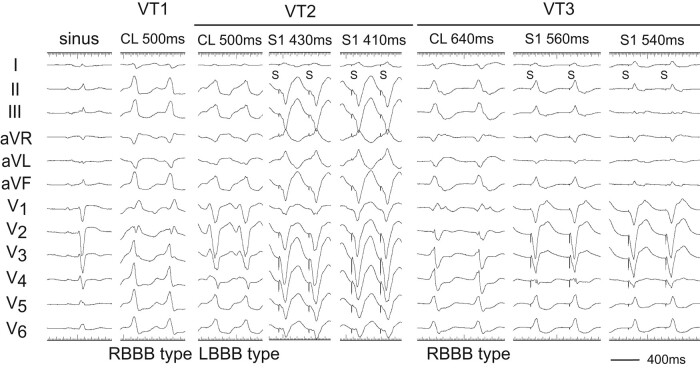
Twelve-lead ECGs of sinus rhythm and ventricular tachycardias (VTs: VT1, VT2, and VT3). After 1st RFCA, the VT1 changed to VT3. During the VT2 and VT3, pacing from the right ventricular apex (RVA) demonstrated constant and progressive fusion. S, stimulus.

A 70-year-old man was transferred for out-of-hospital cardiopulmonary arrest due to ventricular fibrillation and received an implantable cardioverter-defibrillator (ICD) 8 years ago. He was referred for palpitation to our hospital with a VT of left bundle branch block (LBBB) configuration and inferior axis deviation with a heart rate of 115 b.p.m., which was below the VT zone of the ICD. The VT was recurrent, so he had 1st radiofrequency catheter ablation (RFCA). Two types of VTs with right bundle branch block (RBBB) and LBBB morphologies originating from the LV summit were recognized (*Figure 1*; VT1 and VT2). The cycle length of two VTs was ∼500 ms. The VT1 was terminated with RFCA at the left coronary cusp (LCC). However, 9 months later, the VT recurred, so he had 2nd RFCA. The cycle length of the VT was 640 ms with some changes of the LBBB morphology (VT3) (*Figure 1*).

**Figure 2:**
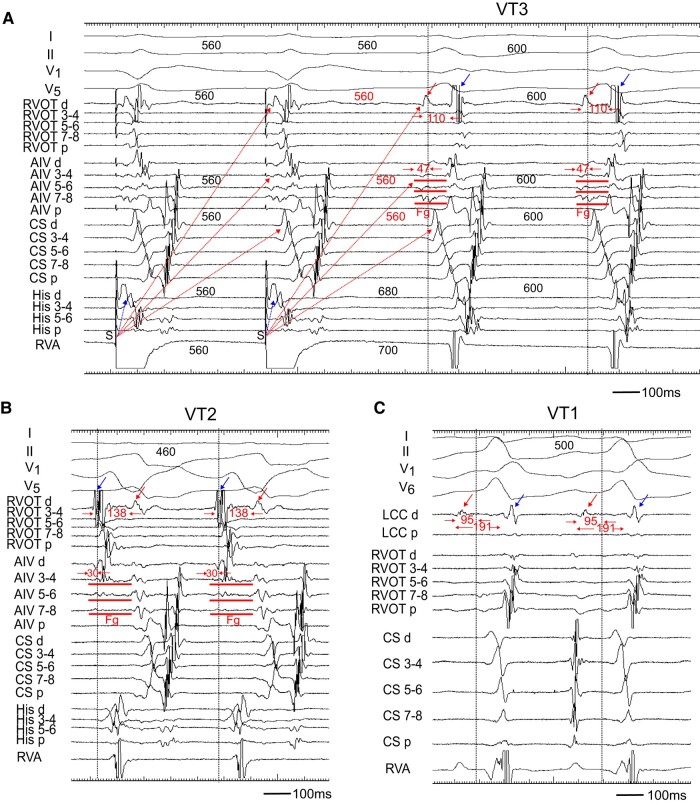
(*A*) Entrainment pacing during the VT3. The AIV catheter recorded a fragmented potential (Fg). During burst pacing from the RVA, the Fg and ventricular potential in the CS catheter activated orthodromically (red dotted arrow), while that in the His catheter was activated antidromically (blue dotted arrow). The distal electrode of the RVOT (RVOT d) recorded a far field potential (red arrow), which was also activated orthodromically (red dotted arrow). The conduction time between the far field (red arrow) and near field (blue arrow) potential was 110 ms. (*B*) During the VT2, the RVOT d recorded the far field potential (red arrow) and near field potential (blue arrow). The sequence of these potentials was reversed as compared to that during the VT3, and the conduction time between two potentials was 138 ms. (*C*) During the VT1, the distal electrode of left coronary cusp (LCC d) also recorded near field (blue arrow) and far field (red arrow) potentials. The conduction time between two potentials was 191 ms. Each dotted vertical line indicates onset of the QRS complex. Shown are the surface ECGs, leads I, II, V1, V5, and V6, and intracardiac electrograms recorded from the right ventricular outflow tract (RVOT), anterior intraventricular vein (AIV), coronary sinus (CS), His bundle (His), right ventricular apex (RVA), and left coronary cusp (LCC) catheter. Fg, fragmented potential; d, distal electrodes; p, proximal electrodes; S, stimulus.

Two decapolar catheters were positioned in the right ventricular outflow tract (RVOT) and in the His bundle-Right ventricular apex (RVA), respectively. Another decapolar catheter with an inner lumen was inserted from the internal jugular vein and placed in the coronary sinus, and a 2 Fr micro-catheter was positioned in the anterior intraventricular vein (AIV) through the lumen. The electrograms in the AIV catheter showed fragmentation (Fg) that preceded the QRS onset by 47 and 30 ms during the VT3 and VT2 (*Figure 2A* and *B*). Pacing from the RVA entrained these two VTs with constant and progressive fusion (*Figure 1*). During the entrainment pacing with a stimulus interval of 560 ms, the fragmented potentials in the AIV catheter were activated orthodromically and those in the His bundle were activated antidromically (*Figure 2A*). During the VT2, a far field potential, which appeared to be intramural potential, was recorded 138 ms later than a near field potential in distal electrodes of the RVOT (*Figure 2B*), whereas during the VT3, the sequence of these potentials was reversed with a conduction time of 110 ms between two potentials (*Figure 2A*), indicating intramural VT substrate.^2^ Moreover, a similar potential was observed during the VT1 in the distal electrodes of a catheter placed in the LCC at the 1st session (*Figure 2C*). These observations indicated the two VTs originated from the LV summit with a re-entrant mechanism, the exit of which was the right ventricle for VT2 and left ventricle for VT3.

**Figure 3:**
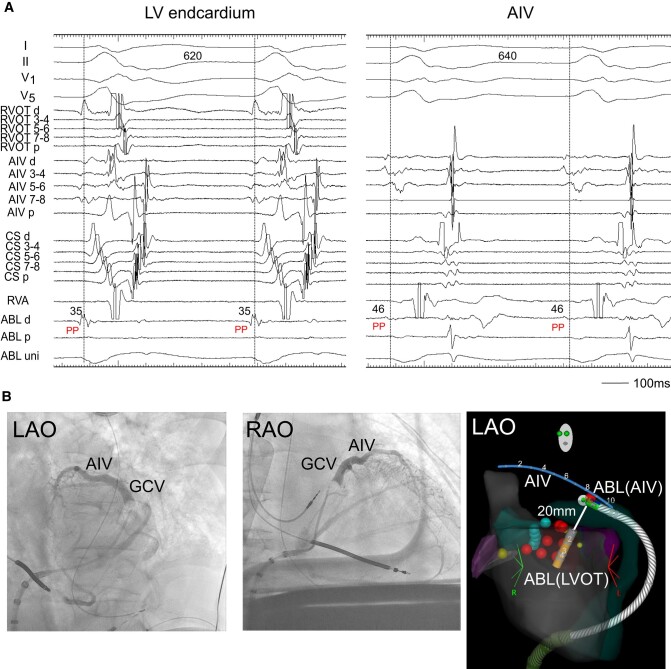
(*A*) Radiofrequency catheter ablation. During the VT2, the ABL, which was located below the LCC, recorded the pre-systolic potential (PP). The PP was also observed at the ABL placed in the AIV. ABL, ablation catheter; PP, pre-systolic potential. Other abbreviations are the same as in *Figure 2*. (*B*) Coronary venography showed AIV and great cardiac vein (GCV). (*C*) Two ablation catheters and an AIV catheter during the bipolar ablation are shown in the electroanatomic mapping image. Bipolar ablation was performed between the left ventricular endocardium (LVOT) and AIV. The distance between the tips of two catheters was 20 mm. ABL (LVOT), an ablation catheter in the left ventricular endocardium; ABL (AIV), an ablation catheter in the AIV.

Radiofrequency catheter ablation was neither effective at the LV endocardium just below the aortic sinus cusp, where the electrogram occurred 35 ms earlier from the onset of QRS complex during the VT3 (*Figure 3A*, left), nor at the RVOT, where a good pace-map of the VT2 was obtained (not showed). So, an irrigated ablation catheter was inserted to the AIV, where the electrogram preceded the onset of QRS by 46 ms (*Figure 3A*, right). At this site, RFCA with an energy of 25 W terminated the VT3. However, the VT3 was still inducible. We thus applied bipolar radiofrequency ablation between the LV endocardium and AIV. We used the T-cable to create a bipolar radiofrequency ablation circuit using the standard unipolar ablation equipment:^3^ A 3.5 mm tip irrigated catheter (FlexAbility™, SE, Abbott) in the LV endocardium that was connected to the ground/impedance port, and another 3.5 mm tip irrigated catheter (ThermoCool SmartTouch, Biosense-Webster, CA, USA) in AIV, which was connected to the active/ablation port. The impedance in the AIV catheter was high. Therefore, we raised the upper limit up to 250 Ω and increased an irrigation flow rate (from 15/min to 28 mL/min). We then delivered an initial energy of 15 W and gradually increased it up to 25 W with careful monitoring in case of sudden impedance drop. Decrease in the impedance during the energy delivery for 75 s was 25 Ω (*Figure 3B*). After three bipolar RF applications, the VTs became non-inducible.

## Discussion

A non-re-entrant mechanism, such as abnormal automaticity or triggered activity, is considered to be operative in the non-sustained VT/premature ventricular contractions originating from the LV summit area. This is because the majority occurred spontaneously or was induced during isoproterenol infusion, but could not be induced by programmed stimulation.^4^ In contrast, some patients had idiopathic sustained LV summit VTs with multiple morphologies, i.e. LBBB and RBBB configurations, which could be induced by programmed stimulation.^5,6^ The preferential conduction to the RVOT and LVOT from a single intramural origin might have produced the multiple VTs.^6^ However, proof of the mechanism of sustained VTs originating from the LV summit area is lacking, probably because of the intramural or subepicardial origin that is inaccessible to the mapping catheter.

In our case, during the VT2 and VT3, the fragmented potential that preceded the QRS onset was observed in the AIV catheter. By entrainment pacing from the RVA, this fragmentation and the far field potential in the RVOT were activated orthodromically, whereas the ventricular electrogram in the His bundle area were activated antidromically (*Figure 2A*). This phenomenon can be translated into the constant fusion and progressive fusion (*Figure 1*), thereby confirming a re-entrant mechanism. Interestingly, we also observed a double potential, which was a far field and near field potential, in the distal electrodes of a catheter placed in the RVOT and LCC. During the VT1 and VT3 with RBBB morphology, the far field potential was preceded to the near field potential, whereas during the VT2 with LBBB morphology, the sequence of these potentials was reversed. The conduction time between these two potentials was more than 100 ms, which indicated intramural VT substrate.^2,7^ For this reason, we needed to performed a bipolar ablation to eliminate the VTs.

## Data Availability

The data underlying this article are available in the article.

## Lead author biography

Masahiro Toba graduated from Toyama University in 2011, and he was a senior resident in the Department of Cardiovascular Medicine at Sapporo City General Hospital from 2013–16. Currently, he is a staff cardiologist at the same hospital. His area of interest is cardiac electrophysiology including catheter ablation of ventricular arrhythmias.

**Consent:** The patient consented to the publication of his case in the scientific literature in accordance with COPE guidelines.

**Funding:** None declared.
